# Disparities in Meeting USPSTF Breast, Cervical, and Colorectal Cancer Screening Guidelines Among Women in the United States

**DOI:** 10.5888/pcd18.200315

**Published:** 2021-04-15

**Authors:** Gabriel A. Benavidez, Anja Zgodic, Whitney E. Zahnd, Jan M. Eberth

**Affiliations:** 1 Department of Epidemiology and Biostatistics, Arnold School of Public Health, The University of South Carolina, Columbia, South Carolina; 2 Rural and Minority Health Research Center, Arnold School of Public Health, The University of South Carolina, Columbia, South Carolina

## Abstract

**Introduction:**

Many sociodemographic factors affect women’s ability to meet cancer screening guidelines. Our objective was to examine which sociodemographic characteristics were associated with women meeting US Preventive Services Task Force (USPSTF) guidelines for breast, cervical, and colorectal cancer screening.

**Methods:**

We used 2018 Behavioral Risk Factor Surveillance System data to examine the association between sociodemographic variables, such as race/ethnicity, rurality, education, and insurance status, and self-reported cancer screening for breast, cervical, and colorectal cancer. We used multivariable log-binomial regression models to estimate adjusted prevalence ratios and 95% CIs.

**Results:**

Overall, the proportion of women meeting USPSTF guidelines for breast, cervical, and colorectal cancer screening was more than 70%. The prevalence of meeting screening guidelines was 6% to 10% greater among non-Hispanic Black women than among non-Hispanic White women across all 3 types of cancer screening. Women who lacked health insurance had a 26% to 39% lower screening prevalence across screening types than women with health insurance. Compared with women with $50,000 or more in annual household income, women with less than $50,000 in annual household income had a 3% to 8% lower screening prevalence across all 3 screening types. For colorectal cancer, the prevalence of screening was 7% less among women who lived in rural counties than among women in metropolitan counties.

**Conclusion:**

Many women still do not meet current USPSTF guidelines for breast, cervical, and colorectal cancer screening. Screening disparities are persistent among socioeconomically disadvantaged groups, especially women with low incomes and without health insurance. To increase the prevalence of cancer screening and reduce disparities, interventions must focus on reducing economic barriers and improving access to care.

**Summary**

**What is already known on this topic?**

Early cancer detection and early treatment initiation increase the chances of survival. Social and environmental context often influences the ability of women to obtain preventive health services such as cancer screening.

**What is added by this report?**

We highlighted the consistently strong association between financial and economic barriers and not meeting cancer screening guidelines among socioeconomically disadvantaged women.

**What are the implications for public health practice?**

Interventions to promote cancer screening should target uninsured women and either provide free screening services or connect them with resources/services that may reduce the cost of screening.

## Introduction

Approximately 40% of new cancer diagnoses and 25% of cancer deaths among women each year are attributed to 3 types of cancer, all of which are amenable to early detection through screening: breast, colorectal, and cervical cancer (1). These cancers have 5-year survival rates at or greater than 90% if diagnosed at a localized stage (1). Because of high survival rates for breast, cervical, and colorectal cancers when detected early, programs such as the National Breast and Cervical Cancer Early Detection Program (2) and the Colorectal Cancer Control Program (3), which provide screening to low-income, uninsured, and underinsured populations, were developed to increase uptake of screening and subsequent follow-up. Although colorectal cancer screening rates increased from 2000 to 2015 as a result in part of increased use of noninvasive screening methods, the proportion of eligible women being screened for cervical and breast cancer decreased nationally by 4.3% and 3.0%, respectively (4).

Racial/ethnic minority populations, women of low socioeconomic status, and women residing in rural areas have had worse cancer survival outcomes than their counterparts (5). From 2013 to 2017, non-Hispanic Black women died of cervical cancer (3.4 per 100,000), breast cancer (27.6 per 100,000), and colorectal cancer (18.5 per 100,000) at higher rates than any other racial/ethnic group (6). Higher mortality among socioeconomically disadvantaged groups is partly due to receiving a diagnosis at a later stage of disease (5). Rural disparities in screening uptake are often attributed to lack of access to screening services and longer travel distances for care (7). The national declines in breast and cervical cancer screening (4) are likely exacerbated among groups that are already socioeconomically disadvantaged and medically underserved, and this exacerbation further widens mortality gaps (8).

Identifying characteristics associated with not meeting cancer screening guidelines could enhance surveillance of possible disparities among groups of people who have historically been economically or socially marginalized. Understanding these factors, whether modifiable or nonmodifiable, will help guide public health efforts, resource allocation, and policies. The objective of our study was to describe the sociodemographic characteristics associated with women meeting US Preventive Services Task Force (USPSTF) guidelines for breast, cervical, and colorectal cancer screening.

## Methods

We used data from the 2018 Behavioral Risk Factor Surveillance System (BRFSS). BRFSS is the largest annual nationally representative telephone survey of the noninstitutionalized US population on health-related risk behaviors, health conditions, and use of preventive health services (9). A full description of BRFSS survey methodologies is published elsewhere (9). In 2018, BRFSS surveyed 437,436 people across all 50 states, the District of Columbia, Guam, and Puerto Rico, with a 53.3% response rate among landline users and a 43.4% response rate among cellular telephone users (10). BRFSS includes core modules that ask questions about screening examinations for breast and cervical cancer, prostate cancer, and colorectal cancer. For this analysis, we used only records for breast, cervical, and colorectal cancer screening among women who identified as residing in any of the 50 US states or the District of Columbia.

### Dependent and independent variables

We examined 3 dependent variables: 1) meeting current breast cancer screening guidelines, 2) meeting current cervical cancer screening guidelines, 3) meeting current colorectal cancer screening guidelines. The USPSTF guidelines recommend that women aged 50 to 74 at average risk be screened for breast cancer by biennial mammography (11). For colorectal cancer, USPSTF guidelines recommend that people aged 50 to 75 at average risk of colorectal cancer be screened by using any of the following methods and frequencies: colonoscopy every 10 years, flexible sigmoidoscopy every 5 years, or yearly stool-based tests (11). USPSTF recommends screening for cervical cancer every 3 years with cervical cytology alone in women aged 21 to 29. For women aged 30 to 65, the USPSTF recommends screening every 3 years with cervical cytology alone, every 5 years with high-risk human papillomavirus testing alone, or every 5 years with high-risk human papillomavirus testing in combination with cytology (11). On the basis of questions on age, types of screenings performed, and when screenings took place, BRFSS computes variables that categorize women’s status for meeting each USPSTF guideline (12).

The Healthy People 2020 framework categorizes the social determinants of health in 5 areas: 1) economic stability, 2) education, 3) social and community context, 4) health and health care, and 5) neighborhood and built environment (13). On the basis of this framework, we used the following sociodemographic variables: annual household income, based on previous BRFSS-generated categories (<$25,000, $25,000 to <$35,000, $35,000 to <$50,000, or ≥$50,000), education (<high school diploma, high school diploma, some college, or college degree), location of residence (metropolitan county, micropolitan county, or rural county as determined by the National Center for Health Statistics’ Urban–Rural Classification Scheme for Counties [14]), health insurance coverage (some form of health insurance or no form of health insurance), employment status (employed, unemployed, or retired), avoidance of medical care because of cost in the past year (yes or no), and race/ethnicity (Hispanic, non-Hispanic Black, non-Hispanic White, or “other” (Asian, Native Hawaiian/Other Pacific Islander, and American Indian/Alaska Native). We categorized age according to USPSTF screening guidelines: for cervical cancer, 3 age groups (21–39, 40–49, and 50–65); for breast cancer and colorectal cancer, 2 groups (50–64 and 65–75).

### Statistical analysis

We produced weighted frequencies for all sociodemographic factors. We then used weighted Wald χ^2^ tests to compare differences in breast, cervical, and colorectal cancer screening by study factors. Accounting for survey weights and nesting (patients nested within states), we constructed mixed-effect log-binomial regression models for each cancer screening subtype. We used log-binomial regression to produce prevalence ratios (PR) and adjusted PRs instead of odds ratios because of the high prevalence of our outcome variables (15). We ran univariate followed by multivariable log-binomial regression models, treating sociodemographic factors as fixed effects and including a random intercept to account for state-to-state variation. We considered the complex survey design structure in our model building. As outlined by Carle (16) and Rabe-Hesketh and Skrondal (17), we rescaled the weights provided by BRFSS at the participant level to reduce the risk of biased estimates in our multilevel model. As described by Goldstein (18) and West et al (19), we created new state-level weights. The use of weights at each level of our mixed-effects model helps generalize our findings beyond our BRFSS sample (19). We generated 95% CIs and used a significance level of .05 throughout our analysis. We used SAS version 9.4 (SAS Institute, Inc) for all statistical analyses. 

## Results

Of the women who responded to the question on breast cancer screening, 56.6% were aged 65 to 75, 72.1% were non-Hispanic White, and 83.3% lived in metropolitan counties (Table 1). Of the women who responded to the question on cervical cancer screening, 48.0% were aged 21 to 39, 60.2% were non-Hispanic White, and 86.7% lived in metropolitan counties. Of the women who responded to the question on colorectal cancer, 56.4% were aged 65 to 75, 72.8% were non-Hispanic White, and 83.2% lived in metropolitan counties. Overall, 78.8%, 80.0%, and 71.3% of eligible women reported meeting USPSTF breast, cervical, and colorectal cancer screening guidelines, respectively (Table 2). For all 3 screening types, in unadjusted analyses, we found significant differences in the proportion of women meeting guidelines by race/ethnicity, annual household income, education, employment status, health insurance status, and reporting medical cost as a barrier to seeking health care (Table 2).

**Table 1:** Selected Sociodemographic Characteristics of Women Who Responded to Behavioral Risk Factor Surveillance System Screening Module, by Cancer Type, 2018^a^

Variable	Breast Cancer (n = 108,746)	Cervical Cancer (n = 105,096)	Colorectal Cancer (n = 109,940)
**Age, y^b^**			
21–39	—	48.0	—
40–49	—	21.3	—
50–65	—	30.7	—
**Age, y^b^**			
50–64	43.4	—	43.6
65–75	56.6	—	56.4
**Race/ethnicity**			
Non-Hispanic White	72.1	60.2	72.8
Non-Hispanic Black	11.2	12.7	11.0
Hispanic	10.4	18.1	10.0
Other^c^	6.3	9.0	6.2
**County type**			
Metropolitan	83.3	86.7	83.2
Micropolitan	9.1	7.7	9.2
Rural	7.5	5.5	7.6
**Annual household income, $**			
<25,000	26.8	26.5	26.8
25,000 to <35,000	9.6	9.4	9.8
35,000 to <50,000	12.6	11.8	12.6
≥50,000	51.0	52.3	50.8
**Education**			
<High school diploma	11.9	10.8	11.7
High school diploma	26.2	22.3	26.4
Some college	33.2	32.1	33.1
College degree	28.7	34.8	28.7
**Current employment status**			
Employed	53.0	60.0	53.1
Unemployed	10.6	8.6	11.7
Retired	36.4	41.4	36.2
**Have some form of health insurance**			
Yes	93.7	86.9	93.9
No	6.3	13.1	6.1
**Avoided medical care because of cost in past year**			
Yes	11.7	16.6	11.4
No	88.3	83.4	88.6

Abbreviation: — , not applicable.

a Source: Centers for Disease Control and Prevention (9). Cancer screening groups are not mutually exclusive. All values are percentages.

b Age categories were created according to screening eligibility criteria defined by the US Preventive Services Task Force.

c Consists of Asian, Native Hawaiian/Other Pacific Islander, and American Indian/Alaska Native.

**Table 2:** Proportion of Women Meeting USPSTF Guidelines by Selected Sociodemographic Factors, Behavioral Risk Factor Surveillance System, 2018^a^

Variable	Breast Cancer Screening	*P* Value^b^	Cervical Cancer Screening	*P* Value^b^	Colorectal Cancer Screening	*P* Value^b^
**Meet screening guidelines**	78.8	—	80.0	—	71.3	—
**Age, y^c^**						
21–39	—	—	79.2	<.001	—	—
40–49	—		83.0		—	
50–65	—		79.3		—	
**Age, y^c^**						
50–64	76.0	<.001	—	—	61.0	<.001
65–75	81.0		—		78.6	
**Race/ethnicity**						
Non-Hispanic White	78.1	<.001	80.0	<.001	72.8	<.001
Non-Hispanic Black	83.9		84.8		73.1	
Hispanic	78.9		80.3		60.8	
Other^d^	77.5		71.2		67.1	
**County type**						
Metropolitan	79.5	<.001	80.4	<.001	71.9	<.001
Micropolitan	76.1		76.9		70.0	
Rural	74.6		75.9		67.1	
**Annual household income, $**						
<25,000	72.3	<.001	74.3	<.001	64.1	<.001
25,000 to <35,000	75.1		76.7		70.8	
35,000 to <50,000	78.1		80.3		72.2	
≥50,000	83.0		84.9		75.4	
**Education**						
<High school diploma	73.1	<.001	74.5	<.001	59.2	<.001
High school diploma	76.9		75.1		69.0	
Some college	78.6		79.2		72.7	
College degree	83.3		85.4		76.8	
**Employment status**						
Employed	80.1	<.001	82.9	<.001	67.7	.005
Unemployed	74.8		77.5		64.9	
Retired	72.5		75.2		64.8	
**Have some form of health insurance**						
Yes	80.5	<.001	82.2	<.001	73.4	<.001
No	54.5		65.0		39.4	
**Avoided medical care because of cost in past year**						
Yes	64.0	<.001	71.7	<.001	56.4	<.001
No	80.8		81.6		73.3	

Abbreviations: — , not applicable; USPSTF, US Preventive Services Task Force.

a Source: Centers for Disease Control and Prevention (9). All values are percentages unless otherwise indicated.

b All *P* values derived by using weighted Wald χ^2^ tests; significance set at *P* < .05.

c Age categories were created according to screening eligibility criteria defined by USPSTF.

d Consists of Asian, Native Hawaiian/Other Pacific Islander, and American Indian/Alaska Native.

### Breast cancer screening

In the adjusted mixed-effects log-binomial models, we found significant differences in meeting breast cancer screening guidelines by sociodemographic factors (Table 3). Compared with women aged 50 to 64, women aged 65 to 75 had a 3% (adjusted PR = 1.03; 95% CI, 1.02–1.05) higher prevalence. The prevalence was higher among non-Hispanic Black (adjusted PR = 1.10; 95% CI, 1.07–1.13) and Hispanic (adjusted PR = 1.08; 95% CI, 1.04–1.13) women than among non-Hispanic White women. The prevalence was 7% (adjusted PR = 0.93; 95% CI, 0.90–0.96) lower among women with an annual household income of less than $25,000 than among women with an annual household income of $50,000 or more. The prevalence among retired women was 3% less (adjusted PR = 0.97; 95% CI, 0.95–0.99) than that of employed women. Women reporting having no form of health insurance coverage had a 26% lower prevalence (adjusted PR = 0.74; 95% CI, 0.68–0.79) than those with some form of health insurance. Women who reported avoiding medical care because of cost had a 15% lower prevalence (adjusted PR = 0.85; 95% CI, 0.81–0.89) than women not avoiding medical care. The prevalence in micropolitan (adjusted PR = 0.99; 95% CI, 0.96–1.01) and rural counties (adjusted PR = 0.98; 95% CI, 0.94–1.01), however, did not differ significantly from the prevalence in metropolitan counties, and the prevalence was no different between women with less than a college degree and women with a college degree.

**Table 3:** Unadjusted and Adjusted Prevalence Ratios (PRs), of Meeting Current USPSTF Screening Guidelines, Behavioral Risk Factor Surveillance System, 2018^a^

Variables	Breast Cancer		Cervical Cancer		Colorectal Cancer	
	Unadjusted PR (95% CI)	Adjusted PR (95% CI)^b^	Unadjusted PR (95% CI)	Adjusted PR (95% CI)^b^	Unadjusted PR (95% CI)	Adjusted PR (95% CI)^b^
**Age, y^c^**						
21–39	—	—	1 [Reference]	1 [Reference]	—	—
40–49	—	—	1.05 (1.02–1.07)	1.02 (1.00–1.04)	—	—
50–65	—	—	1.00 (0.98–1.02)	0.98 (0.96–0.99)	—	—
**Age, y^c^**						
50–64	1 [Reference]	1 [Reference]	—	—	1 [Reference]	1 [Reference]
65–75	1.29 (1.24–1.34)	1.03 (1.02–1.05)	—	—	1.06 (1.05–1.08)	1.23 (1.19–1.28)
**Race/ethnicity**						
Non-Hispanic White	1 [Reference]	1 [Reference]	1 [Reference]	1 [Reference]	1 [Reference]	1 [Reference]
Non-Hispanic Black	1.07 (1.04–1.10)	1.10 (1.07–1.13)	1.06 (1.03–1.08)	1.06 (1.04–1.08)	1.01 (0.97–1.04)	1.07 (1.03–1.12)
Hispanic	1.01 (0.97–1.06)	1.08 (1.04–1.13)	1.01 (0.99–1.03)	1.05 (1.03–1.07)	0.83 (0.77–0.88)	0.97 (0.92–0.99)
Other^d^	0.98 (0.95–1.01)	1.01 (0.95–1.06)	0.89 (0.86–0.92)	0.90 (0.87–0.93)	0.90 (0.88–0.93)	0.95 (0.91–1.00)
**County type**						
Metropolitan	1 [Reference]	1 [Reference]	1 [Reference]	1 [Reference]	1 [Reference]	1 [Reference]
Micropolitan	0.97 (0.94–0.99)	0.99 (0.96–1.01)	0.96 (0.91–1.00)	0.98 (0.95–1.02)	0.97 (0.95–1.00)	0.98 (0.94–1.02)
Rural	0.95 (0.92–0.98)	0.98 (0.94–1.01)	0.93 (0.91–0.96)	0.97 (0.94–1.00)	0.93 (0.90–0.95)	0.93 (0.88–0.98)
**Annual household income, $**						
<25,000	0.87 (0.85–0.89)	0.93 (0.90–0.96)	0.87 (0.85–0.89)	0.94 (0.91–0.96)	0.85 (0.81–0.89)	0.92 (0.89–0.96)
25,000 to <35,000	0.91 (0.89–0.93)	0.92 (0.89–0.95)	0.90 (0.88–0.93)	0.95 (0.92–0.98)	0.94 (0.91–0.97)	0.96 (0.91–0.99)
35,000 to <50,000	0.94 (0.93–0.96)	0.95 (0.92–0.97)	0.94 (0.93–0.95)	0.97 (0.96–0.99)	0.96 (0.93–0.99)	0.95 (0.92–0.99)
≥50,000	1 [Reference]	1 [Reference]	1 [Reference]	1 [Reference]	1 [Reference]	1 [Reference]
**Education**						
<High school diploma	0.88 (0.85–0.92)	0.98 (0.94–1.01)	0.87 (0.80–0.95)	0.97 (0.90–1.04)	0.77 (0.73–0.82)	0.88 (0.83–0.94)
High school diploma	0.93 (0.92–0.94)	0.99 (0.96–1.01)	0.88 (0.86–0.90)	0.95 (0.92–0.97)	0.90 (0.89–0.92)	0.92 (0.89–0.95)
Some college	0.95 (0.93–0.96)	0.97 (0.95–1.00)	0.93 (0.91–0.95)	0.97 (0.94–0.99)	0.95 (0.93–0.97)	0.96 (0.92–0.99)
College degree	1 [Reference]	1 [Reference]	1 [Reference]	1 [Reference]	1 [Reference]	1 [Reference]
**Employment status**						
Employed	1 [Reference]	1 [Reference]	1 [Reference]	1 [Reference]	1 [Reference]	1 [Reference]
Unemployed	0.94 (0.92–0.96)	0.98 (0.95–1.00)	0.94 (0.92–0.95)	0.97 (0.95–1.00)	0.96 (0.93–0.99)	0.97 (0.94–1.01)
Retired	0.91 (0.89–0.94)	0.97 (0.95–0.99)	0.91 (0.89–0.93)	0.96 (0.94–0.98)	0.96 (0.94–0.99)	1.02 (1.00–1.05)
**Have some form of health insurance**						
Yes	1 [Reference]	1 [Reference]	1 [Reference]	1 [Reference]	1 [Reference]	1 [Reference]
No	0.68 (0.64–0.72)	0.74 (0.68–0.79)	0.79 (0.75–0.83)	0.83 (0.79–0.88)	0.54 (0.49–0.59)	0.61 (0.56–0.66)
**Avoided medical care because of cost in past year**						
Yes	0.80 (0.77–0.82)	0.85 (0.81–0.89)	0.88 (0.86–0.89)	0.94 (0.92–0.97)	0.77 (0.73–0.81)	0.91 (0.87–0.96)
No	1 [Reference]	1 [Reference]	1 [Reference]	1 [Reference]	1 [Reference]	1 [Reference]

Abbreviations: — , not applicable; USPSTF, US Preventive Services Task Force.

a Source: Centers for Disease Control and Prevention (9). Log-binomial regression was used to estimate prevalence ratios instead of odds ratios to produce less biased measures of association due to the high frequency of the outcome variable (meeting screening guidelines) in this analysis.

b Adjusted log-binomial regression models include age, race/ethnicity, county type, annual household income, education, employment status, health insurance status, and whether the respondent avoided medical care because of cost in the past year.

c Age categories were created according to screening eligibility criteria defined by USPSTF.

d Consists of Asian, Native Hawaiian/Other Pacific Islander, and American Indian/Alaska Native.

### Cervical cancer screening

We found significant differences in meeting cervical cancer screening guidelines by sociodemographic factors in the adjusted weighted mixed-effects log-binomial models (Table 3). We found no difference in prevalence among women aged 40 to 49, compared with women aged 21 to 39, but we found a 2% (adjusted PR = 0.98; 95% CI, 0.96–0.99) lower prevalence among women aged 50 to 65. Compared with non-Hispanic White women, non-Hispanic Black (adjusted PR = 1.06; 95% CI, 1.04–1.08) and Hispanic (adjusted PR = 1.05; 95% CI, 1.03–1.07) women had a higher prevalence. Women at any annual household income level lower than $50,000 had a lower prevalence ranging from 3% to 6%. Compared with women with a college degree, women with some college (adjusted PR = 0.97; 95% CI, 0.94–0.99) and women with a high school diploma (adjusted PR = 0.95; 95% CI, 0.92–0.97) had a lower prevalence. Retired women (adjusted PR = 0.96; 95% CI, 0.94–0.98) had a lower prevalence compared with employed women, but we observed no difference between unemployed women and employed women. Women reporting having no form of health insurance had a 17% lower prevalence (adjusted PR = 0.83; 95% CI, 0.79–0.88) compared with women with some form of health insurance. Women who reported avoiding medical care because of cost had a 6% lower prevalence (adjusted PR = 0.94; 95% CI, 0.92–0.97) than those not avoiding medical care. We observed no significant difference in the adjusted model for women residing in micropolitan (adjusted PR = 0.98; 95% CI, 0.95–1.02) or rural counties (adjusted PR = 0.97; 95% CI, 0.94–1.00) compared with metropolitan counties. 

### Colorectal cancer screening

We found significant differences in meeting colorectal cancer screening guidelines by sociodemographic factors in the adjusted weighted mixed-effects log-binomial models (Table 3). Compared with women aged 50–64, women aged 65 to 75 had a 23% (adjusted PR = 1.23; 95% CI, 1.19–1.28) higher prevalence. Non-Hispanic Black (adjusted PR = 1.07; 95% CI, 1.03–1.12) women had higher prevalence than non-Hispanic White women. In contrast, Hispanic women had a 3% (adjusted PR = 0.97; 95% CI, 0.92–0.99) lower prevalence than non-Hispanic White women. Women in rural counties had a 7% lower prevalence (adjusted PR = 0.93; 95% CI, 0.88–0.98) than women in metropolitan counties; we found no significant difference between women in micropolitan counties and women in metropolitan counties. Women in the lowest income level had an 8% (adjusted PR = 0.92; 95% CI, 0.89–0.96) lower prevalence than women at the highest income level. Compared with women with a college degree, women with less than a college degree had a lower prevalence ranging from 4% to 12%. We found no significant difference between women who were unemployed or retired and women who were employed. Women who reported having no form of health insurance had a 39% lower prevalence (adjusted PR = 0.61; 95% CI, 0.56–0.66) than women with some form of health insurance. Women who reported avoiding medical care because of cost had a 9% lower prevalence (adjusted PR = 0.91; 95% CI, 0.87–0.96) than women not avoiding medical care.

## Discussion

This study examined sociodemographic factors and their association with meeting USPSTF guidelines for breast, cervical, and colorectal cancer screening. Our findings suggest that women currently not meeting screening guidelines share many characteristics. Women who have an annual household income less than $50,000, have less than a college education, live in rural counties, and lack ability to pay for medical care because of cost or lack of health insurance have a lower prevalence of meeting USPSTF guidelines for breast, cervical, and/or colorectal cancer screening than their more socioeconomically advantaged counterparts and women living in metropolitan counties.

Although most of our results demonstrate a lower prevalence of meeting guidelines among people who historically have been medically underserved, the prevalence of meeting guidelines for all screening types was higher among non-Hispanic Black women than among non-Hispanic White women in adjusted models. Analyses of similar nationally representative data sets have produced similar results (4,6). The prevalence of some screenings has been consistently higher among non-Hispanic Black women than among non-Hispanic White women since 1987 (6). In our analysis, Hispanic women also had a higher prevalence of breast and cervical cancer screening than their non-Hispanic White counterparts. However, they had a lower prevalence of colorectal cancer screening, consistent with previous research (6). Reasons for this trend among Hispanic populations are not well understood. Research on culturally specific characteristics among the various Hispanic nationalities is needed to better understand why Hispanic people fall behind other racial/ethnic groups in colorectal cancer screening. Previous data highlight disparities in cancer mortality between racial/ethnic minority groups, especially non-Hispanic Black women and their white counterparts. Our analysis confirms that higher cancer mortality among racial/ethnic minority groups will not be reduced solely by increasing rates of cancer screening. Although preventive screenings and timely diagnosis are important elements of prognosis, they are just 2 elements of many along the cancer care continuum that need to be addressed to eliminate disparities in cancer mortality.

We found that women in rural counties had a lower prevalence of meeting colorectal cancer screenings guidelines than women in metropolitan counties, even after accounting for other sociodemographic characteristics. Health care professionals with specialized training most often perform colorectal cancer screenings by colonoscopy or sigmoidoscopy. Women in rural areas may have a lower prevalence of meeting colorectal cancer screening guidelines because rural areas often have limited access to specialized health care services (20). Additionally, the limited access to health care services in rural areas often means that people living in rural areas must travel long distances to reach areas where advanced health care services are provided (21). These disadvantages in the early detection and treatment of cancer are compounded by the fact that rural residents, on average, have lower incomes than their nonrural counterparts and lower rates of health insurance coverage (22).

We found disparities in meeting USPSTF screening guidelines among economically disadvantaged women, defined in our study as having an annual household income less than $50,000, not having health insurance, or reporting avoiding medical care because of cost. In the past 2 decades, women with an income less than 200% of the federal poverty level have had consistently lower cancer screening rates than women with incomes above 200% of the federal poverty level (6). Women with low incomes, for numerous reasons, are less likely to have a usual source of primary care — where preventive screenings and measures are often discussed and performed. National programs such as the Centers for Disease Control and Prevention–funded Colorectal Cancer Control Program and National Breast and Cervical Cancer Early Detection Program were developed with the primary aim of eliminating cost as a barrier for breast, cervical, and colorectal cancer screening and follow-up. These programs distribute funding to health care centers to provide eligible low-income, underinsured, and uninsured women access to screening, diagnostic, and cancer treatment services. Further work is needed to ensure that these programs are used and expanded. Of women eligible for the National Breast and Cervical Cancer Early Detection Program during 2015–2017, only 6.7% received cervical cancer screening and 15% breast cancer screening services. Furthermore, not all states receive funding from the Colorectal Cancer Control Program. Targeted outreach and awareness of these Centers for Disease Control and Prevention programs and similar programs are needed: our results and the results of previous studies demonstrate a consistently strong association between economic barriers and lack of meeting screening guidelines. Increased use of these programs may reduce cancer mortality among women with persistent economic barriers to care (23).

Overall, sustainable solutions to cancer screening disparities will require large-scale policy changes and smaller-scale health education and awareness campaigns on the importance of cancer screening. Shifting to a single-payer health care system in the US may save an estimated 68,000 lives annually by removing economic barriers to preventive health care such as cancer screening and routine checkups (24). Hendryx and Luo showed an increase in the proportion of low-income women being screened in states that expanded Medicaid under the Affordable Care Act (25). The expansion of Medicaid is likely to have the largest effect in states that have large rural and low-income populations and consistently demonstrate poor health outcomes, such as states in the Southeast (26). Additionally, evidence supports the efficacy of small-scale interventions to increase screening rates in some locations and populations (27). A meta-analysis of randomized controlled interventions designed to increase colorectal cancer screening rates demonstrated that in diverse health care populations, the use of patient navigators, a type of barrier-focused intervention in which trained specialists assist patients in navigating logistical barriers of the cancer screening process, increased screening rates by approximately 20 percentage points (27).

Our study has several strengths. One strength is our mixed-effects modeling approach, which allowed us to account for state-to-state variation in the data. Economic and social structure varies from state to state, and accounting for this variation allowed us to generate less biased PRs. Second, our adjusted models accounted for several potentially confounding variables, but residual confounding may still be present in our PR estimates. Future studies may benefit from incorporating and merging state and county-level variables with BRFSS data to provide better area-level context for more representative estimates. Third, because BRFSS is nationally representative sample, the results of our study are generalizable to women in the general US population. 

Our study also has potential limitations. First, although BRFSS is the nation’s premier surveillance mechanism for health behaviors, BRFSS data are self-reported. Our estimates depend on respondents providing accurate information with minimal recall bias or social desirability bias. However, studies have found these biases not to be associated with self-reported cancer screening adherence (28). Second, BRFSS is a telephone-based survey that limits responses to people with access to a telephone and only people who answer and are willing to participate. However, a new weighting methodology known as raking now allows BRFSS to consider telephone ownership in the weighting process, potentially minimizing bias resulting from telephone-based data collection. Third, our screening prevalence estimates may be overestimated. Comparisons of data from BRFSS and the National Health Interview Survey (NHIS) found that screening prevalence estimates were consistently higher in BRFSS (29). One reason BRFSS screening estimates are higher is that the survey is designed to produce estimates at the state level whereas NHIS is designed to produce estimates at the national level. The aggregation of state-level BRFSS data to generate a national estimate likely biases the estimates upwards. Despite these potential limitations, the absolute difference in estimates between BRFSS and other nationally representative surveys in most cases is small from a surveillance perspective (30).

Efforts have been made to increase the proportion of women who meet cancer prevention screening guidelines. Most women in the US meet USPSTF guidelines, but continued attention needs to be directed toward women who do not. Across all 3 cancer screening types, women facing economic barriers had a consistently lower prevalence of meeting preventive screening guidelines. Interventions and policy changes to reduce economic barriers are expected to increase cancer screening uptake to meet benchmarks such as Healthy People 2030 goals (31). 
